# Species-level view of population structure and gene flow for a critically endangered primate (*Varecia variegata*)

**DOI:** 10.1002/ece3.1119

**Published:** 2014-06-06

**Authors:** Andrea L Baden, Sheila M Holmes, Steig E Johnson, Shannon E Engberg, Edward E Louis, Brenda J Bradley

**Affiliations:** 1 Department of Anthropology, Yale UniversityNew Haven, Connecticut, 06511; 2 Department of Anthropology, Hunter College of City University of New YorkNew York, 10065; 3 Department of Anthropology, University of CalgaryCalgary, Alberta, Canada; 4 Grewcock's Center for Conservation and Research – Omaha's Henry Doorly Zoo and AquariumOmaha, Nebraska, 68107

**Keywords:** Conservation genetics, dispersal, genetic diversity, lemur, Madagascar

## Abstract

Lemurs are among the world's most threatened mammals. The critically endangered black-and-white ruffed lemur (*Varecia variegata*), in particular, has recently experienced rapid population declines due to habitat loss, ecological sensitivities to habitat degradation, and extensive human hunting pressure. Despite this, a recent study indicates that ruffed lemurs retain among the highest levels of genetic diversity for primates. Identifying how this diversity is apportioned and whether gene flow is maintained among remnant populations will help to diagnose and target conservation priorities. We sampled 209 individuals from 19 sites throughout the remaining *V. variegata* range. We used 10 polymorphic microsatellite loci and ∼550 bp of mtDNA sequence data to evaluate genetic structure and population dynamics, including dispersal patterns and recent population declines. Bayesian cluster analyses identified two distinct genetic clusters, which optimally partitioned data into populations occurring on either side of the Mangoro River. Localities north of the Mangoro were characterized by greater genetic diversity, greater gene flow (lower genetic differentiation) and higher mtDNA haplotype and nucleotide diversity than those in the south. Despite this, genetic differentiation across all sites was high, as indicated by high average *F*_ST_ (0.247) and Φ_ST_ (0.544), and followed a pattern of isolation-by-distance. We use these results to suggest future conservation strategies that include an effort to maintain genetic diversity in the north and restore connectivity in the south. We also note the discordance between patterns of genetic differentiation and current subspecies taxonomy, and encourage a re-evaluation of conservation management units moving forward.

## Introduction

Lemurs are among the world's most endangered mammals (IUCN 2013). Currently, 93 of 103 lemur taxa (90%) are classified as at least vulnerable and the number of species listed as Critically Endangered (i.e., at extremely high risk of extinction in the wild) has tripled since 2008 (Schwitzer et al. 2013). Understanding the genetic structure of these threatened populations, particularly those that exist in degraded or fragmented habitats, is not only an urgent priority for conservation efforts (Schwartz et al. 2006; Frankham 2010), but also relevant to developing environmental and climate change models (e.g., Ikeda et al. 2012).

Because of its unique biota (Ganzhorn et al. 2001), Madagascar is routinely identified as a global conservation priority (Myers et al. 2000; Robinson 2006). Since the 1950s, more than half of Madagascar's remaining forest cover has been cleared and forest edges have quadrupled (Harper et al. 2007). In fact, some authorities estimate as much as 85–90% of primary vegetation has already been lost (e.g., Myers et al. 2000). Land use practices, including logging, mining, and slash and burn agriculture (tavy), continue to threaten Madagascar's unique flora and fauna (McConnell 2002; Kull 2004; Mittermeier et al. 2010). Such practices have led to habitat loss and fragmentation that may potentially restrict or eliminate gene flow between subpopulations and result in rapid population declines and genetic bottlenecks. As a consequence, populations may be susceptible to reduced genetic diversity via drift and inbreeding depression (Nei 1975). This in turn poses significant threats to small, isolated populations by limiting their genotypic and phenotypic flexibility and long-term resilience to environmental changes (Madsen et al. 1999; Reed and Frankham 2003). Determining the distribution of genetic diversity and whether subpopulations are in migratory contact can highlight important dispersal corridors, as well as identify isolated populations, thereby suggesting priority areas for conservation (Schwartz et al. 2006).

The critically endangered black-and-white ruffed lemur (*Varecia variegata*) provides an ideal case study with which to investigate these relationships. Ruffed lemurs are medium-sized (3–4 kg; Baden et al. 2008), arboreal obligate frugivores that live in large, spatially dispersed social groups, also known as communities (Morland 1991; Vasey 2003; Baden and Gerber in review). This species is among the most frugivorous of the Malagasy primates (74–90%, Balko 1998), making it particularly sensitive to habitat degradation; in fact, ruffed lemurs are among the first to disappear in the face of habitat loss (White et al. 1995). Furthermore, their boom-bust reproductive strategy (i.e., long, synchronous interbirth intervals followed by “booms” in reproduction, whereby all breeding females within a community bear litters of 2–3 offspring; Baden et al. 2013) and slow life histories result in a relatively low reproductive rate (Baden et al. 2013). Previous population estimates suggest that fewer than 10,000 *V. variegata* individuals remain (Mittermeier et al. 2010). However, the species’ patchwork distribution throughout Madagascar's remaining eastern rainforest corridor makes accurate population estimates difficult and suggests that the true population size of this taxon could be far less (Irwin et al. 2005). These remaining *V. variegata* individuals are fragmented into several geographically distinct localities with limited potential for reproductive contact and unknown population structure. Individuals within these localities are under continued threat from habitat loss and fragmentation, and more recently bushmeat hunting, particularly in the northern distribution of their range (Golden 2009).

**Figure 1:**
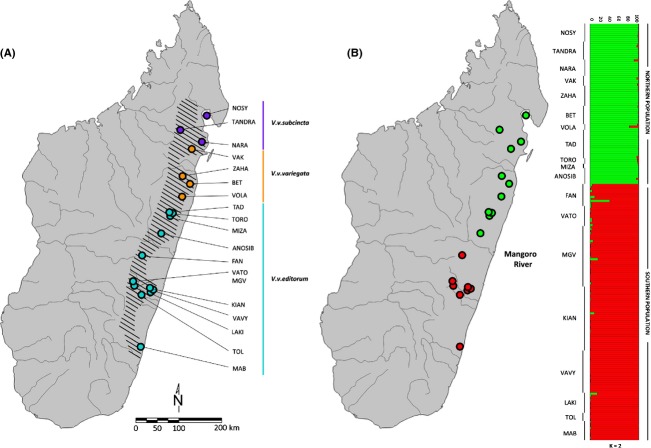
Map illustrating the estimated species distribution and current subspecies designations (A) and results from population structure analysis (B) illustrating the proportional membership (*Q*) of each ruffed lemur in the two clusters identified. Animals are each represented by a single horizontal bar. Locality of origin is indicated to the left of each individual (see Table 1 for full site names). Structure results are consistent using both biparentally and maternally inherited markers.

Previous studies have found evidence of genetic isolation and population decline (Holmes et al. 2013), as well as low haplotype diversity (Wyner et al. 1999) at several ruffed lemur sites. Nevertheless, recent comparative genomics research indicates that ruffed lemurs have among the highest measures of genetic diversity for primates (Perry et al. 2013), implying that this critically endangered lemur species may still harbor considerable genetic variation throughout parts of its range. Given the risk of rapid decline and isolation of these populations, there is an immediate need to understand how this genetic variation is distributed and what geophysical and/or anthropogenic barriers influence gene flow among localities. Furthermore, it is important to evaluate the extent to which existing subspecies designations (*V. v. subcincta*, *V. v. variegata, V. v. editorum*; Table 1; Fig. 1) are concordant with population structure to help gauge conservation priorities and inform captive management programs.

**Table 1:** Sampling localities, subspecies designations and sample sizes used in this study

Site name	Site code	Subspecies^2^	Latitude	Longitude	*n*	Nm	Nf
Nosy Mangabe S.R.^1^	NOSY	*V. v. subcincta*	S15°30′11.7″	E049°45′30.5″	9	4	5
Marotandrano S.R.	TANDRA	*V. v. subcincta*	S16°16′8.25″	E048°49′08.3″	9	4	5
Mananara Nord N.P.	NARA	*V. v. subcincta*	S17°34′36.5″	E049°57′20.8″	8	4	4
Ambatovaky S.R.	VAK	*V. v. variegata*	S16°49′01.4″	E049°16′24.5″	5	4	1
Zahamena N.P., S.N.R.	ZAHA	*V. v. variegata*	S17°29′21.0″	E048°44′50.0″	10	3	7
Betampona S.N.R.	BET	*V. v. variegata*	S17°55′87.1″	E049°12′20.0″	9	5	4
Mangerivola S.R.	VOLA	*V. v. variegata*	S18°14′11.4″	E048°54′27.5″	3	1	2
Mantadia Andasibe N.P.	TAD	*V. v. editorum*	S18°48′49.0″	E048°25′47.8″	14	9	5
Torotorofotsy U.F.	TORO	*V. v. editorum*	S18°50′07.7″	E048°21′03.9″	3	1	2
Maromizaha U.F.	MIZA	*V. v. editorum*	S18°58′30.2″	E048°27′43.5″	2	1	1
Anosibe an'ala C.F.	ANOSIB	*V. v. editorum*	S19°13′76.8″	E048°16′86.0″	8	3	5
Fandriana U.F.	FAN	*V. v. editorum*	S20°23′40.2″	E047°38′09.8″	11	5	6
Vatoharanana (Ranomafana N.P.)	VATO	*V. v. editorum*	S21°14′90.0″	E047°25′26.6″	10	5	5
Mangevo (Ranomafana N.P.)	MGV	*V. v. editorum*	S21°22′22.8″	E047°26′59.1″	30	14	16
Kianjavato U.F.	KIAN	*V. v. editorum*	S21°21′43.4″	E047°50′54.3″	32	18	14
Vatovavy U.F.	VAVY	*V. v. editorum*	S21°24′20.0″	E047°56′26.0″	21	10	11
Lakia U.F.	LAKI	*V. v. editorum*	S21°28′52.5″	E047°53′29.0″	10	4	6
Tolongoina U.F.	TOL	*V. v. editorum*	S21°35′30.0″	E047°29′06.0″	4	2	2
Manombo S.R.	MAB	*V. v. editorum*	S23°01′69.5″	E047°43′84.1″	11	6	5
Total sample					209	103	106

UF, unclassified forest; CF, classified forest; SR, Special Reserve; SNR, Strict Nature Reserve; NP, National Park, *n*: total sample; Nm: number of males sampled; Nf: number of females sampled.

1 Introduced population.

2 Subspecies designations following Andrainarivo et al. (2009), Mittermeier et al. (2010).

As long-term gene flow and the apportionment of genetic diversity are linked to short-term natal dispersal, we also test hypotheses regarding sex-biased dispersal in this species. Previous studies found some behavioral evidence of male transfer between communities (Morland 1991; Balko 1998), and females are generally considered the philopatric sex (Kappeler 1997; but see Erhart and Overdorff 2008). Accordingly, we predicted that black-and-white ruffed lemur communities would consist of unrelated males and closely related females, although some molecular evidence from red ruffed lemurs (*V. rubra*) suggests that both sexes disperse (Razakamaharavo et al. 2010).

Here, we describe the population genetic structure and dispersal patterns of black-and-white ruffed lemurs (*V. variegata*) as inferred from microsatellite markers and mitochondrial DNA sequence variation. Our analysis is unusually in that our samples were collected across the extent of the species’ range and thereby provides a species-level view of genetic apportionment for a critically endangered primate.

## Methods

### Sample collection and storage

We sampled a total of 209 adult individuals (103 males, 106 females) from 19 localities from across the existing *V. variegata* range (*n* = 2–32 individuals per locality; Table 1; Fig. 1). Distances between localities ranged from six to 860 km. To obtain these data, field assistants from Omaha's Henry Doorly Zoo and Aquarium (OHDZA) and the Madagascar Biodiversity Partnership (MBP) immobilized study individuals with 10 mg/kg estimated body weight of Telazol® (Fort Dodge Animal Health, IA), administered by Dan-Inject (Børkop, Denmark) Model JM CO_2_-powered projection rifle and 9 mm disposable Pneu-Darts™ (Williamsport, PA). Whole blood (1 mL/kg) samples were collected from the femoral vein and stored at room temperature in 5 mL of lysis buffer solution (0.1 mol/L Tris-HCl pH, 8.0, 0.1 mol/L EDTA, 0.01 mol/L NaCl, and 0.5% w/v SDS) (Seutin et al. 1991) until they were banked in a −80°C freezer at the OHDZA. Sample collection occurred under veterinary supervision and followed a strict protocol outlined by Glander (1993). All capture procedures occurred during nonreproductive seasons in the absence of infants and dependent offspring.

Additional noninvasive sampling occurred in two sites (Kianjavato: *n* = 20 individuals, and Vatovavy, *n* = 11 individuals). In these cases, SMH, MBP, and field assistants collected fecal samples from ruffed lemurs that had been previously collared for individual identification. Researchers and assistants collected 2–5 samples per lemur and removed seeds from fecal samples prior to preservation in RNAlater® (Life Technologies, Grand Island, NY) at a ratio of 1 mL feces to 5 mL RNAlater®. Samples were kept at room temperature for 15–105 days until transported to OHDZA, where they were stored at −20°C.

Importantly, samples were collected from only adult individuals to minimize the chance of sampling parent/offspring pairs, and samples were collected across different social groups. Furthermore, our analysis of relatedness and sex-biased dispersal did not indicate clusters of close relatives within sampling locales.

Immobilizations, handling, sample collections, and export/import protocols adhered to and were approved by the OHDZA's Institutional Animal Care and Use Committee (IACUC #97-001), Stony Brook University IACUC (#2005-20081449), University of Calgary Life and Environmental Sciences Animal Care Committee (BI11R-15), Malagasy wildlife authorities, Convention on International Trade in Endangered Species regulations, and US Fish & Wildlife Service.

### DNA extraction

Total genomic DNA was extracted from blood and fecal samples using standard nucleic acid extraction kits (QIAamp® DNA Mini Kit & DNA Stool Mini Kit; QIAGEN, Valencia, CA) following the manufacturer's protocols. Ten microsatellite loci (Louis et al. 2005), which regularly and reliably amplified fecal DNA in initial tests, were used to genotype all individuals (see Table S1).

#### DNA from blood

Microsatellite loci were amplified in 25 *μ*L reactions consisting of 2 *μ*L DNA template (50–80 ng), 12.5 *μ*L QiagenHotStarTaq Master Mix and 10 *μ*mol/L of each primer. Amplification conditions were as follows: initial denaturation at 95°C for 15 min; 35 cycles of 30 sec at 94°C, 40 sec at 54 to 60°C (see Louis et al. 2005), 1 min at 72°C, and a final extension of 7 min at 72°C. The 5′ end of the forward primer was fluorescently labeled, and amplification products were separated using capillary electrophoresis (ABI 3730*xl* Genetic Analyzer).

#### DNA from feces

For the low-quality fecal DNA extracts, we carried out PCR amplifications in a 25 *μ*L volume with 4 *μ*L template (20–50 ng), 12.5 *μ*mol/L of each primer, 1.5 mmol/L MgCl_2_, 200 *μ*mol/L dNTP, 10 mmol/L Tris (pH 8.3), 50 mmol/L KCl, and 0.5 U Taq DNA polymerase (Promega; Madison, WI). Thermal cyclers profiles were as follows: 35 cycles of 30 sec at 95°C, 30 sec at 54 to 60°C, and 30 sec at 72°C with a 10 min final extension phase at 72°C (see also Table S1).

### Microsatellite genotyping

Amplification products were separated using capillary electrophoresis (ABI 3730xl Genetic Analyzer), and alleles were sized relative to an internal size standard (ROX-500) using Gene Mapper software v. 4.0 (Applied Biosystems, Foster City, CA). To detect and avoid allelic dropout, multiple PCR replicates were performed according to the concentration of DNA in each sample (Morin et al. 2001). Final genotypes were scored based on multiple independent reactions (Taberlet et al. 1996); all heterozygotes were confirmed by a minimum of two separate reactions and homozygous genotypes were typically confirmed via five amplifications. DNA yields from fecal samples were substantially lower than from blood samples, thereby sometimes requiring greater numbers of replicates (range 3–11) to confirm homozygous genotypes (as in Morin et al. 2001). The observed probability of identity (P_ID_; Paetkau and Strobeck 1994) for all markers was 9.51 × 10^−15^, demonstrating the very low probability that two individuals would share the same multilocus genotype.

### mtDNA sequencing

For a geographically representative subset of individuals (*n* = 159), we amplified the D-loop or control region of the mitochondrial DNA (mtDNA) using primers dLp5 (Baker et al. 1993) and DLp1.5 (Wyner et al. 1999). We generated 555 bp fragments using 50 ng of DNA and the following conditions: 94°C for 4 min, 35 cycles of 94°C for 30 sec, 47°C for 45 sec, 72°C for 45 sec and 72°C for 10 min.

To exclude potential amplification of nuclear insertions, we subsequently generated the PCR products with a quick, efficient species independent technique derived from the degenerate oligonucleotide primed PCR method (DOP-PCR; Telenius et al. 1992). Adapting the long products from low-quantity DOP-PCR methodology (LL-DOP-PCR), we verified sequence data generated from overlapping segments for the D-loop, COII, 12S rRNA, and PAST PCR fragments. Amplifications were carried out on a MBS Satellite 0.2G Thermal Cycler (Thermo Electron Corporation; Waltham, MA) and verified by electrophoresing samples on a 1.2% agarose gel. We purified samples using the QIAquick PCR purification kit (Qiagen), cycle-sequenced them using a BigDye Terminator v3.1 Cycle Sequencing Kit (Applied Biosystems) and generated sequences with a 7% polyacrylamide gel by an ABI 377 automated sequencer (Applied Biosystems).

Individual sequences were analyzed, edited and aligned using Sequencher 4.9 (Gene Corp; Ann Arbor, MI). Final alignment of all sequences was performed using ClustalX software (Thompson et al. 1997) and checked by eye. Notably, each sample yielded a single clear, unambiguous sequence (i.e., no evidence of heteroplasmy), further indicating that amplicons were not a mixture of mitochondrial and nuclear targets.

### Population genetic analyses

#### Genetic diversity

All loci were tested for the presence of null alleles using Micro-Checker (van Oosterhout et al. 2004) and linkage disequilibrium using Genepop v.3.4 (Raymond and Rousset 1995). Departures from equilibrium were evaluated with 10,000 permutations. We used GenoDive v.2.0b23 (Meirmans and Van Tienderen 2004) to calculate measures of genetic diversity, including the number of alleles per locus (*n*_*A*_), the mean number of alleles per sampling locality (*MNA*), allelic richness per locality (*AR*), and observed (*H*_O_) and expected (*H*_E_) heterozygosities (Nei 1978) for each sampling locality. To account for differences in sample size (Kalinowski 2004), allelic richness (*AR*) was standardized to the smallest sample size in the dataset using rarefaction implemented in HP-Rare 1.0 (Kalinowski 2005). Finally, we estimated Wright's *F*_IS_ (a measure of deviation from Hardy–Weinberg equilibrium) according to Weir and Cockerham (1984) and tested populations with a sufficient sample set for significant deviations from equilibrium with 10,000 permutations.

#### Inferring population genetic structure

We used two methods to infer population structure from our sample of 209 ruffed lemur microsatellite genotypes. We first used the model-based Bayesian clustering method implemented in Structure v2.3.4 (Pritchard et al. 2000) to infer the optimal number of genetic populations (*K*) as suggested by the microsatellite data. This method uses the Markov Chain Monte Carlo (MCMC) approach to group individuals into *K* populations based on their multilocus genotypes without prior information regarding their sampling localities (i.e., USEPOPINFO was not specified). We also calculated the fractional membership of individuals within each population (*Q*). We evaluated the hypotheses *K* = 1–22, the number of sampling locations plus 3, following Evanno et al. (2005). From a pilot study, we determined that 50,000 iterations of burn-in followed by 100,000 iterations of Markov Chain Monte Carlo (MCMC) were sufficient to allow convergence of parameters prior to data collection; longer burn-in or MCMC did not result in significant changes in our results (data not shown). Because different runs produced different likelihood values, we carried out 20 runs for each value of *K* assuming correlated allele frequencies and admixture. Using the admixture model allowed us to estimate the number of natural genetic clusters and detect historical population admixture (Falush et al. 2003; Ostrowski et al. 2006). We identified the most likely number of populations (*K*) using the Δ*K* method (Evanno et al. 2005) implemented in Structure Harvester v0.6.93 (Earl and vonHoldt 2012). Using this method, optimum *K* is identified by the highest value of Δ*K*, or the second order rate of change in the likelihood of *K,* which corresponds to the most pronounced genetic subdivision present within the data. For the chosen value of *K*, we averaged *Q* across the 20 independent runs. As the Δ*K* method generally identifies the highest level of structure in the dataset, we took a two-step approach. First, we identified the most likely number of clusters within the overall sample (*n* = 209). We then ran subsequent analyses within each of the *K* clusters from the original run following Evanno et al. (2005) to evaluate whether further substructure existed.

To corroborate Structure results, we performed a principal coordinates analysis (PCoA) with a standard genetic distance matrix (Nei 1978) using GenAlEx v.6.5 (Peakall and Smouse 2012).

Finally, we performed a follow-up exclusion test (Cornuet et al. 1999) in Geneclass 2.0 (Piry et al. 2004). Using population simulations, we statistically tested whether one or more of the sampled localities could be ruled out as the area of origin for each individual. The probability of individual genotypes coming from each locality was calculated by comparing individual genotypes to 10,000 simulated individuals per locality. We used the default criteria for computation parameters and selected the Paetkau et al. (2004) simulation method.

Results from the Bayesian cluster assignments and PCoA guided subsequent analyses. First, we examined microsatellite population genetic structure with a locus-by-locus Analysis of Molecular Variance (AMOVA, Excoffier et al. 1992) implemented in GenoDive. We used permutation tests of 10,000 iterations to examine the distribution of genetic variation at four hierarchical levels: among populations (i.e., *K* clusters), among sampling localities within populations, among individuals within sampling localities, and within individuals. Distances were calculated using the Infinite Alleles Model (*F*_ST_ analog).

We also compared allelic diversity at each microsatellite marker between the inferred *K* populations using the log-likelihood G test of genotypic variation implemented in Genepop (Rousset 2008). Significance was calculated using 10,000 randomizations not assuming HWE (Goudet et al. 1996). Finally, we performed pairwise tests for population differentiation (*F*_ST_) in GenoDive. Significance was calculated using 10,000 randomizations not assuming HWE and corrected for multiple comparisons (Bonferroni adjusted *P* = 0.0004).

Dispersal or migration barriers can hinder gene flow, thereby increasing genetic distance, even in cases where populations are not geographically distant (Liu et al. 2009; Quéméré et al. 2009). Thus, to investigate the relationship between genetic distance among sampling localities and their geographic distances, we performed tests of spatial autocorrelation (isolation-by-distance) implemented in GenAlEx. We used the Geographic Distance Matrix Generator v.1.2.3 from the American Museum of Natural History (http://biodiversity-informatics.amnh.org/open_source/gdmg/) to calculate pairwise geographic distances (in km) between all sampling localities based on their decimal degree coordinates. This matrix was then compared with a matrix of Nei's genetic distances (described previously) using Mantel matrix correlations. Significance was evaluated based on 9,999 permutations.

To examine mtDNA population genetic structure, we used a standard AMOVA for haplotype data implemented in GenAlEx. We used permutation tests of 9,999 iterations, this time at three hierarchical levels (within individual comparisons are not possible in haplotype AMOVAs including only one locus): among populations, among sampling localities within populations, and among individuals within sampling localities. We calculated nucleotide and haplotype diversity using DnaSP v5 (Librado and Rozas 2009) and inferred haplotype networks of mtDNA sequences using a median-joining algorithm (Bandelt et al. 1999) implemented in Network v.2.2 (Fluxus, Clare, Suffolk, UK). Epsilon (*ε*) was set equal to zero and variable sites were weighted equally. Finally, we performed pairwise tests for population differentiation (Φ_ST_) in GenoDive. Significance was calculated using 10,000 randomizations not assuming HWE and corrected for multiple comparisons (Bonferroni adjusted *P* = 0.0004).

### Inferences of population dynamics

#### Sex-biased dispersal

We evaluated whether dispersal was sex-biased following methods described by Goudet et al. (2002) and implemented in Fstat 2.9.3 (Goudet 2001). For both males and females, we estimated and compared the following measures: levels of inbreeding (*F*_IS_); average relatedness (*R*); population differentiation (*F*_ST_); mean Assignment Indices (*m*AIc); and variance in Assignment Indices (*v*AIc). *F*_IS_ represents a measure of how well genotype frequencies within a population match expectations of Hardy–Weinberg Equilibrium (Hartl and Clark 1997) and can be used to detect a reduction in heterozygosity that is typically caused by population substructure. Because the dispersing sex in a population often includes a combination of both immigrants and residents, the admixture of these two populations should lead to a resultant heterozygote deficiency (and a positive *F*_IS_) within the dispersing sex. The dispersing sex should also have lower average relatedness (*R*) among postdispersal aged members of a population than members of the more philopatric sex because dispersal reduces the likelihood that relatives are living in close association (Greenwood 1980; Goudet et al. 2002; but see Lukas et al. 2004). Consequently, *F*_ST_, or the measurable proportion of genetic variance attributable to among-population differentiation, should be lower in the dispersing sex because the less philopatric sex should be less differentiated in its allele frequencies among populations (i.e., increased gene flow yields fewer genetic differences between populations in the dispersing sex) (Hartl and Clark 1997). Finally, members of the dispersing sex should show significantly lower mean Assignment Indices, but higher variance than members of the more philopatric sex (Lawson Handley and Perrin 2007). Assignment Indices are statistics that summarize the likelihood that an individual's multilocus genotype originated in the population from which it was sampled and can be used to test for differences in the mean values (*m*AIc) and the variance (*v*AIc) of assignments between the sexes. These indices can then be standardized, subtracting the population mean AI from each individual's AI (Favre et al. 1997), such that animals with positive “corrected” assignment indices (AIc) are those which are more likely to have been born in the population, while immigrant genotypes are less likely to occur in the sample and should therefore have negative AIc values (Goudet et al. 2002). In sum, compared with the philopatric sex, the dispersing sex is predicted to have (1) positive *F*_IS_ values, (2) lower average relatedness, (3) lower values of *F*_ST_, (4) lower mean assignment scores (*m*AIc) and 5) greater variance in assignment (*v*AIc). We calculated two-tailed *P*-values using 10,000 randomizations, where sex was randomly assigned to genotypes while keeping the sex ratio and group identity constant thereby producing a null distribution (see Table 4). We also compared the average number of haplotypes shared by males and by females using a two-tailed Student's *t*-test.

#### Bottleneck analyses

Finally, we tested for genetic signatures of recent population decline using Bottleneck software (Luikart and Cornuet 1998; Piry et al. 1999). We used a Wilcoxon signed-ranks test to compare observed and expected heterozygosity at mutation-drift equilibrium (H_Eeq_) because of its robusticity to small sample sizes (<30) and small numbers of loci (<20) (Piry et al. 1999). As the mutation model underlying the real data is never known and is likely to change from locus to locus, we used three models (IAM: infinite alleles model; SMM: stepwise mutation model; and TPM: two-phase mutation model) in parallel to assess whether departures from mutation-drift equilibrium were robust under all models or sensitive to model changes (e.g., Goossens et al. 2006). Default parameters were used and significance was evaluated with 10,000 replications.

## Results

### Genetic diversity

All ten loci were polymorphic with 4–13 alleles each (Table S1). We pooled individuals from across sampling localities and found no evidence of significant linkage disequilibrium across markers. Deviations from Hardy–Weinberg equilibrium (HWE) were present in 15 of 190 possible locus-site combinations (10 loci × 19 sites), likely reflecting the relatively small sample size for some localities (Table S1). Of these, five loci had positive values and two loci had negative values of *F*_IS_ and no locus stood out as an outlier. For these reasons, all loci were kept in the analysis. Sampling localities averaged between 2.2–4.7 alleles (Table S2).

### Population genetic structure

From our first Structure analysis of 209 individuals, we identified two genetic clusters, as indicated by the highest value of Δ*K* (Fig. 1; Fig. S1). These results were the same whether we used biparentally or maternally inherited markers. From the microsatellite analyses, Cluster 1 consisted exclusively of individuals from the eleven northern-most sampling localities (80 of 209 members; Table 1; Fig. 1), and Cluster 2 comprised only individuals sampled from the eight southernmost sampling localities (129 of 209 members; Table 1; Fig. 1). It is important to note that these genetic clusters did not correspond to the current taxonomy of the species that separates *V. variegata* into three separate subspecies (Table 1; Fig. 1).

We repeated the analysis with each of the *K* = 2 clusters separately following Evanno et al. (2005) and found that Cluster 1 (northern sampling localities) and Cluster 2 (southern sampling localities) could each be further subdivided into *K* = 2 clusters (Figs. S2 and S3). In both cases, sampling localities appear to cluster primarily by latitude (i.e., geographic location) and perhaps also according to habitat connectivity. Sampling localities from the original Cluster 1 (North) grouped into *K* = 2 distinct subpopulations (Subcluster 1.1: Nosy, Tand, Nara; Subcluster 1.2: Tad, Toro, Miza, Anosib). Sites located between these localities (Vak, Zaha, Bet, Vola) shared varying degrees of proportional membership with each of the *K* = 2 clusters. Furthermore, we observed a second, albeit small peak at *K* = 4 (Fig. S2). Subdivision still occurred mainly according to geographic proximity. The four southernmost populations (Tad, Toro, Miza, Anosib) still clustered into a single population. However, with the exception of Nosy, northern sampling localities (Tandra, Nara, Vak, Zaha, Bet, Vola) exhibited substantial admixture and shared varying levels of proportional membership among the remaining *K* = 3 clusters. It is interesting to note, however, that animals from Nosy, an island population whose founders were introduced in the 1930s (Petter and Peyreiras 1970), cluster almost exclusively with individuals from Nara, perhaps suggesting their provenance (Fig. S3).

Similar patterns of substructure were detected among populations in the south. Localities within the original Cluster 2 (South) grouped into *K* = 2 subpopulations. The two sampling localities from within Ranomafana National Park (Vato, Mgv) clustered together with the northernmost (Fan) and southernmost localities (Mab) in Subcluster 2.1, while the fragmented (and geographically proximate) habitats of Kian, Vavy, and Laki clustered together in a second subcluster (Subcluster 2.2). Tol exhibited substantial admixture of the two.

**Figure 2:**
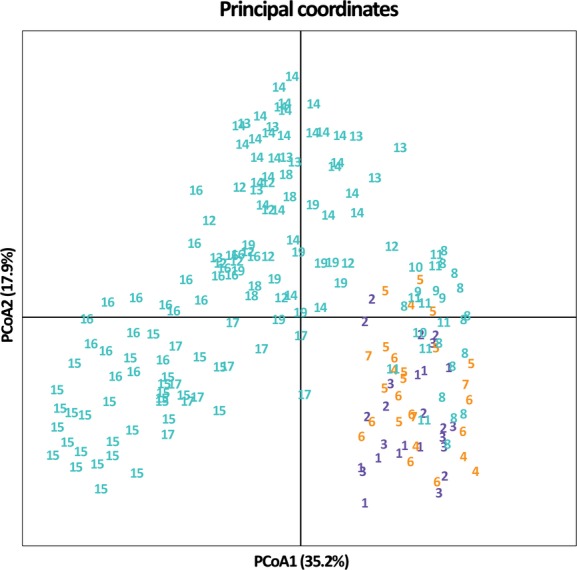
Principle coordinate analysis (PCoA). Data points are represented by numbers that correspond to sampling locality (1–19) and are color coded according to current subspecies assignments (purple: *Varecia variegata subcincta*; orange: *V. v. variegata*; blue: *V. v. editorum*).

The principle coordinate analysis (PCoA; Fig. 2) corroborated Structure results, in that it showed a clear separation along axis 1 (PCo1) that grouped sampling localities into two clusters. Clusters showed minimal overlap and appeared to separate according to geographic location. Sampling localities north of the Mangoro River clustered together (bottom right), as did sampling localities to the south of the river (top left). This component accounted for 35.2% of the total molecular variance. Another 17.9% of the variance was explained by Axis 2 (PCo2), which seemed to primarily separate sampling localities within the south. It is interesting to note that, again, the genetic clustering did not correspond to subspecies status (Fig. 2).

**Figure 3:**
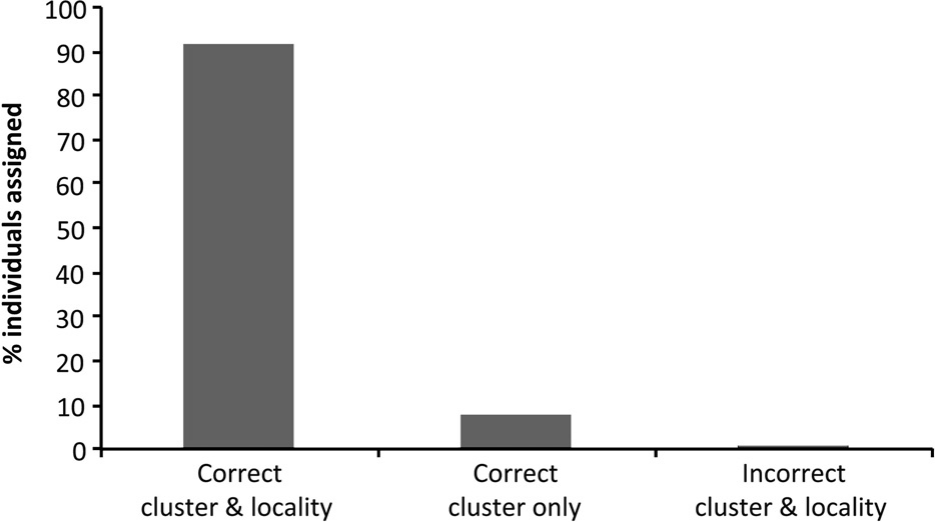
Distribution of highest probability assignments as determined using the resampling procedure in GENECLASS.

From the microsatellite data, an exclusion test with resampling accurately assigned 91.4% of individuals to both their correct sampling locality and cluster when considering the locality of highest probability (Fig. 3). In the 8.6% of cases where individuals were incorrectly assigned, most were assigned to localities belonging to the same cluster (7.7%). Less than 1% of individuals were incorrectly assigned to both sampling locality and cluster/population. However, in 97.9% of the correct assignments, additional localities other than that with highest probability could not be ruled out as the source population. This was true for individuals in the northern cluster (73 of 73 individuals; 100%) and the southern cluster (114 of 118; 96.6%).

Allelic diversity within the two clusters ranged from four to 13 alleles (mean = 8.9); four to 11 alleles (mean = 8) in the north and three to 13 (mean = 6.6) in the south (Table 2). Genotypic differentiation between the two clusters was highly statistically significant overall (*P* < 0.0001), as well as for each of the 10 markers individually (Table 2). Results from the locus-by-locus AMOVA revealed pronounced levels of structure in the microsatellite data (Table 3). Although the highest percentage of variation was within individuals, due to high levels of heterozygosity, there was significant variation apportioned to all hierarchical levels of *V. variegata*.

**Table 2:** Comparison of number of alleles between northern (*n* = 80) and southern (*n* = 129) populations of *V. variegata*. *P*-values correspond to 10,000 randomizations of log-likelihood G tests of population differentiation for each marker

	No. alleles			
		
Marker	North	South	Total	*G* test *P*-value
51HDZ20	11	8	13	<0.001
51HDZ25	5	3	5	<0.001
51HDZ204	4	3	4	<0.001
51HDZ247	10	7	10	<0.001
51HDZ560	9	7	10	<0.001
51HDZ598	8	7	9	<0.001
51HDZ691	11	11	13	<0.001
51HDZ790	4	4	5	<0.001
51HDZ816	10	8	10	<0.001
51HDZ988	8	8	10	<0.001
Mean	8.0	6.6	8.9	

**Table 3:** Locus-by-locus AMOVA of 10 microsatellite markers for 209 *V. variegata* individuals and standard AMOVA for haplotype data of mtDNA d-loop sequences (*n* = 159). *P* is based on 10,000 permutations. df = degrees of freedom, SS = sum of squared deviations, MS = mean of squared deviations

Variance component	df	SS	MS	Variation	Proportion of total variation	Statistic	P
Locus-by-locus microsatellite AMOVA							
Among populations (northern and southern)	1	126.97	–	0.543	0.136	*F*_ct_	<0.001
Among sites within populations	17	266.55	–	0.601	0.150	*F*_sc_	<0.001
Among individuals within sites	190	585.79	–	0.233	0.058	*F*_IS_	<0.001
Within individuals	209	547.00	–	2.617	0.655	*F*_IT_	<0.001
Standard mtDNA haplotype AMOVA							
Among populations (northern and southern)	1	253.45	253.454	2.703	0.328	PhiRT	<0.001
Among sites within populations	17	574.06	33.768	3.905	0.474	PhiPR	<0.001
Among individuals within sites	140	227.60	1.626	1.626	0.197	PhiPT	<0.001

Pairwise *F*_ST_ comparisons suggest the same pattern of relationships as the Bayesian methods and are consistent with results from the locus-by-locus AMOVA suggesting significant levels of genetic differentiation both between northern and southern clusters and among sampling localities (Table S3). Overall, genetic differentiation among sampling localities as measured by *F*_ST_ was significant in 101 of 171 cases (Table S3). Pairwise values of *F*_ST_ among sampling localities ranged from 0.002 to 0.442, with a mean of 0.241, though these are viewed with caution given the range of sample sizes (*N* = 2–32, Table 1). Within-cluster *F*_ST_ values were almost always lower than between-cluster comparisons (data not shown). Somewhat contrary to the individual-based analyses, most sampling localities show significant divergence from one another; however, this is more prevalent among southern sampling localities (24 of 28 pairs differed significantly; 85.7%) than among localities in the north (17 of 55 pairs; 30.9%).

In addition, we found strong evidence of isolation-by-distance (i.e., a significant positive correlation between genetic and geographic distance matrices among individuals; *r*^2^ = 0.501 *P* < 0.0001). This same pattern held true when looking among individuals within either of the two clusters (North: *r*^2^ = 0.424, *P* < 0.0001; South: *r*^2^ = 0.428, *P* < 0.0001).

**Figure 4:**
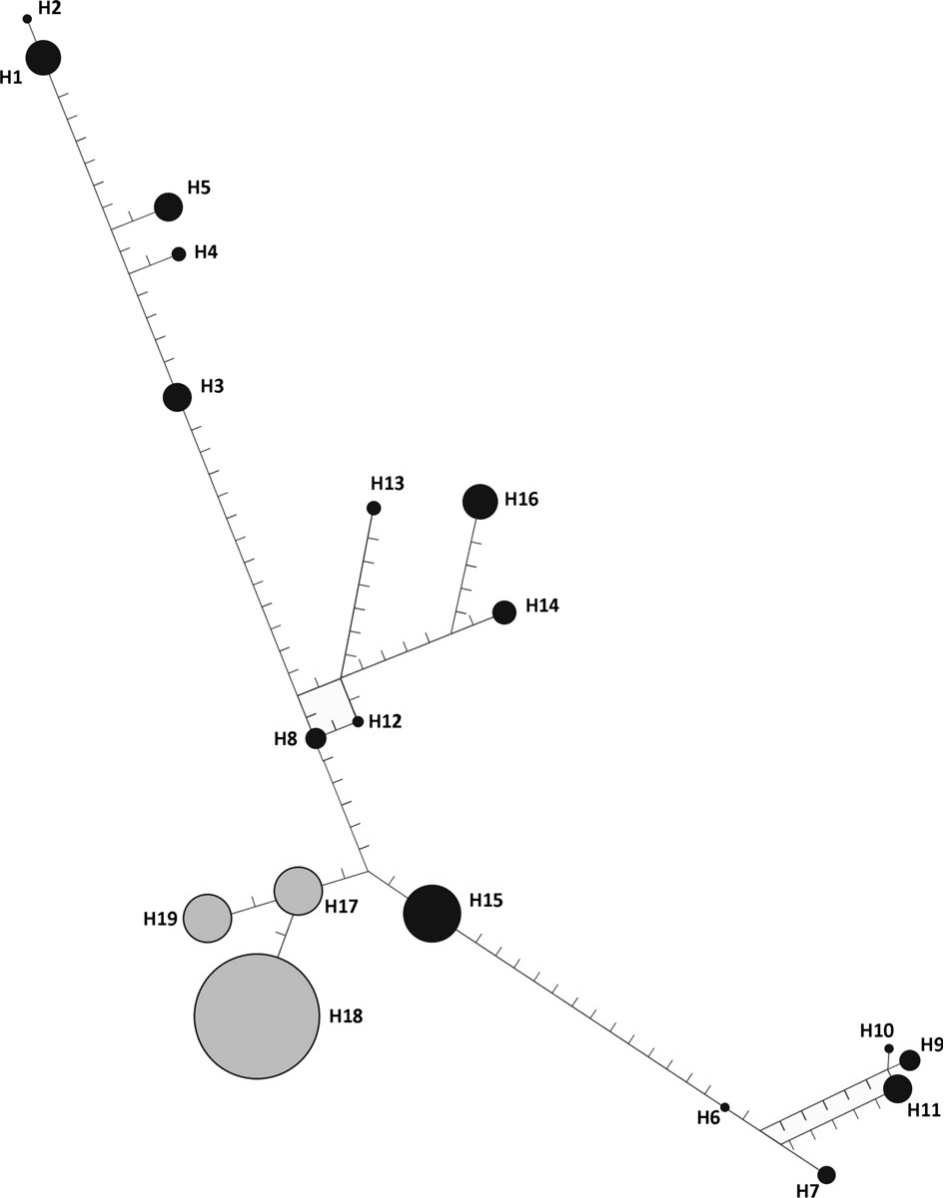
Haplotype networks of *V. variegata* mtDNA d-loop sequences created using a median-joining algorithm implemented in NETWORK. Shading indicates populations identified from STRUCTURE analyses (black = northern; gray = southern). Size of the node corresponds to the frequency of that haplotype among sampled individuals. Internal nodes represent reconstructed median haplotypes. Notches represent nucleotide differences between haplotypes.

Aligning 159 *V. variegata* mtDNA sequences (North *n* = 83; South *n* = 76), we found 19 haplotypes and 44 polymorphic sites (Table S4). Of these 44 single nucleotide polymorphisms (SNPs), there were no fixed differences and no haplotype sharing between northern and southern clusters. The mitochondrial DNA of the northern cluster was much more diverse (43 SNPs; 16 haplotypes) than the southern cluster (2 SNPs; 3 haplotypes). These results were reflected in median-joining network analysis, which again shows clear clustering into northern and southern groups (Fig. 4).

The standard AMOVA for haplotype data shows strong differentiation among all hierarchical levels of the analysis (Table 3). Population differentiation is stronger for mtDNA than microsatellite markers, with variation among populations explaining 33% of the total variation in mtDNA, while only 14% of total variation in microsatellite markers is explained by variation among populations. Similarly, variation among sites within populations explains nearly half (48%) of the total variation in mtDNA, whereas among-site variation explains only 17% in microsatellites (Table 3).

As with measures of pairwise *F*_ST_, comparisons of Φ_ST_ suggest the same pattern of relationships as the Bayesian methods and correspond with results from the standard AMOVA suggesting significant levels of genetic differentiation both within clusters and among sampling localities (Table S5).

### Population dynamics, sex-biased dispersal and gene flow

We did not find evidence of sex-biased dispersal (Table 4). Estimates of *R*, *F*_ST,_ and *F*_IS_ did not differ significantly between males and females, suggesting that both sexes are equally likely to disperse. Although *m*AIc was positive for males (mean = 0.218) and negative for females (mean = −0.216), this difference was not significant. Moreover, variance in AIc was very high for both sexes, and differences were not significant. Furthermore, males and females did not differ significantly in their number of haplotypes across sampling localities (two-tailed Student *t*-test, *P* = 0.369).

**Table 4:** Mean values and tests of sex-biased dispersal using microsatellite data from *n* = 92 adult males and *n* = 93 adult females

		Results			
		
Test	Predicted	Male	Female	Observed	P
*F*_IS_	+	0.264	0.261	=	0.94
*R*	−	0.223	0.216	=	0.79
*F*_ST_	−	0.153	0.148	=	0.77
mAIc	−	0.218	−0.216	+	0.54
vAIc	+	24.853	20.539	+	0.48
Haplotypes	+	1.420	1.260	=	0.37

Predictions based on previous evidence of male-biased dispersal.

Results based on 10,000 randomizations in FSTAT.

Significance in haplotype number tested using a two-tailed Student's *t*-test.

Finally, using Bottleneck, we found evidence of deviations from mutation-drift equilibrium at 5 of 9 sampling localities when either the IAM or TPM models were assumed (Table 5). However, 10 of 19 sampling localities were excluded from this analysis due to small sample sizes.

**Table 5:** Analysis of past population bottleneck events under each of three mutation models

		Mutation model			
			
Site code	*n*	IAM	TPM	SMM	Mode shift
NOSY	9	–	–	–	–
TANDRA	9	–	–	–	–
NARA	8	–	–	–	–
VAK	5	–	–	–	–
ZAHA	10	0.053	0.246	0.500	Shifted
BET	9	–	–	–	–
VOLA	3	–	–	–	–
TAD	14	**0.001**	**0.001**	**0.019**	**Shifted**
TORO	3	–	–	–	–
MIZA	2	–	–	–	–
ANOSIB	8	–	–	–	–
FAN	11	0.065	0.348	0.920	Normal
VATO	10	0.116	0.246	0.652	Normal
MGV	30	**0.000**	**0.001**	0.053	**Shifted**
KIAN	32	0.216	0.500	0.839	Normal
VAVY	21	**0.001**	**0.019**	0.213	Normal
LAKI	10	**0.001**	**0.001**	**0.001**	**Shifted**
TOL	4	–	–	–	–
MAB	11	**0.012**	**0.053**	0.116	**Shifted**
Total	209				

IAM: infinite allele model; TPM: two-phase model; SMM: stepwise mutation model Significant *P*-values (bold) indicate an excess of heterozygosity under each of three mutation models. Mode shift provide qualitative description of shifts from low to medium frequency alleles in a population. Significance calculated using one-tailed Wilcoxon signed-ranks test. *P* < 0.05. Samples with fewer than 10 samples were not included in this analysis.

## Discussion

### Genetic diversity and population structure

Together, our analyses show significant genetic differentiation among sampling localities, with a primary division north and south of the Mangoro River (Figs. 1B, 2 and 4). This division does not, however, correspond to the current taxonomy (Fig. 1A; see below). We also found some degree of substructure within each cluster, though this was more pronounced in southern versus northern sampling localities; in the south, substructure was inconsistent with geographic clustering of sites (Fig. S3). While Structure cannot detect fewer than *K* = 2 genetic clusters, given that PCoA and exclusion analyses both support this same pattern of clustering, we regard *K* = 2 populations as the best-supported hypothesis at this time (see also Evanno et al. 2005).

Localities characterized as forest fragments (i.e., unclassified & classified forests, Table 1) show clear patterns of isolation-by-distance (IBD) both within and between northern and southern clusters. Over time, genetic drift is expected to eliminate patterns of IBD. Therefore, the spatial autocorrelation observed in this study could be indicative of relatively recent interconnectivity among localities via forest corridors (e.g., Ranomafana–Andringitra Corridor; Mittermeier et al. 2005).

Despite high levels of of *F*_ST_ and IBD, sampling localities clustered together, regardless of whether samples derived from forest fragments or national parks within larger forest blocks, and these patterns were true whether we used biparentally or maternally inherited genetic markers. This is in contrast to previous studies that have found comparatively more substructure among fragmented habitats versus continuous forest sites, despite being separated by comparable geographic distances (Olivieri et al. 2008; Oaklander et al. 2010; Schneider et al. 2010; Holmes et al. 2013). Together, our results suggest that while forest fragmentation and habitat loss have increased genetic differentiation among sampling localities, it may have occurred recently enough that genetic differentiation has not yet increased beyond the drift effects of pure isolation-by-distance.

Looking within each of the two genetic clusters, we found that *V. variegata* individuals located in northern sampling localities are characterized by significantly higher allelic diversity, greater genetic and haplotypic diversity, and higher levels of gene flow than *V. variegata* individuals located within the southern cluster of sampling localities. It is possible that different environmental and/or landscape conditions are operating in the two geographic regions to produce these divergent results. Further investigation (e.g., landscape genetic analysis) will help to better understand the patterns observed herein.

Nevertheless, our results clearly indicate that gene flow among localities is limited. Average *F*_ST_ for this species is the highest observed in any lemur study to date (Table 6). Moreover, although ruffed lemurs are distributed over a relatively narrow geographic range (i.e., the eastern rainforest corridor of Madagascar), their level of genetic differentiation is an order of magnitude greater than chimpanzees distributed across all of (western to eastern) equatorial Africa (average *F*_ST_ = 0.014, Langergraber et al. 2011). These results highlight the importance of taking a species-specific approach when identifying potential dispersal barriers (i.e., barriers to some species may not hinder dispersal in others; Baguette and Van Dyck 2007).

**Table 6:** Population differentiation according to *F*_ST_

						Sampling locality					
								
Family	Species	Sample	Marker	Sample *N*	Entire range?	*N*	Min *F*_ST_	Max *F*_ST_	Mean *F*_ST_ ± SD	Inferred K clusters	Primary structuring factors
Daubentoniidae	*Daubentonia madagascariensis*^1^	Tissue	Genomic DNA (666,256 SNPs)	12	No	8	(0.129)	(0.194)	(0.164 ± 0.033)	3	–
Cheirogaleidae	*Microcebus bongolavensis*^2^	Tissue	8 microsats	45	No	3	0.057	0.102	0.076 ± 0.023	1	Rivers & geographic distance
	*Microcebus danfossi*^2^	Tissue	8 microsats	78	No	7	0.025	0.195	0.096 ± 0.049	2	Rivers & geographic distance
	*Microcebus murinus*^3^	Tissue	10 microsats	167	No	3	0.004	0.016	–	1	–
	*Microcebus ravelobensis*^4^	Tissue	8 microsats	187	No	12	−0.002	0. 122	0.052 ± 0.027	3	Road or other (unknown) & geographic distance
	*Microcebus ravelobensis*^2^	Tissue	8 microsats	205	No	8	0.006	0.156	0.072 ± 0.035	2	Rivers & geographic distance
Lepilemuridae	*Lepilemur mustelinus*^5^	Blood	3 enzyme loci	72	No	4	−0.026	0.133	0.055 ± 0.080	–	–
Indriidae	*Propithecus tattersali*^6^	Feces	13 microsats	82	No	3	0.136	0.160	(0.147)	2	Rivers & geographic distance; Road not a barrier
	*Propithecus tattersali*^7^	Feces	13 microsats	230	Yes	9	0.010	0.300	0. 119 ± 0.067	3	Rivers & geographic distance; Road not a barrier
	*Propithecus verreauxi*^8^*	Tissue	7 microsats	77–131	No	10–28	0.024	0.075	0.052	–	–
Lemuridae	*Eulemur cinereiceps*^9^	Tissue	26 microsats	53	No	4	0.020	0.076	0.054 ± 0.019	–	Geographic distance
	*Varecia rubra*^10^	Tissue	15 microsats	32	No	2	–	–	0.077	–	–
	*Varecia variegata*^11^	Blood, feces	16 microsats	55	No	5	0.039 (0.193)	0.291 (0.229)	0.197 ± 0.084 (0.212)	3	–
	*Varecia variegata*^12^	Blood, feces	10 microsats	209	Yes	19	0.002	0.441	0.247 ± 0.094 (0.163)	2	Rivers & geographic distance

1 Perry et al. (2013).

2 Olivieri et al. (2008).

3 Fredsted et al. (2005).

4 Radespiel et al. (2008).

5 Tomiuk et al. (1997).

6 Quéméré et al. (2009).

7 Quéméré et al. (2010).

8 Lawler et al. (2003).

9 Brenneman et al. (2011).

10 Razakamaharavo et al. (2010).

11 Holmes et al. (2013).

12 this study.

*F*_ST_ comparisons are among sampling localities unless otherwise noted. Values in parentheses denote comparisons among *K* inferred clusters.

* Denotes groupwise comparisons within a single population.

### Barriers to gene flow

The northern and southern clustering occurred on either side of the Mangoro River (Fig. 1B), the largest river in eastern Madagascar. These results support the principles of Martin (1972) and are in accordance with long-standing hypotheses regarding Malagasy microendemism and patterns of population structure (e.g., Martin 1972; Wilmé et al. 2006; Craul et al. 2007). Similar patterns have been found among many of the Malagasy strepsirrhines, including other large-bodied, diurnal species of *Propithecus* and *Eulemur* (Ganzhorn et al. 2006). Interestingly, in both of these examples, populations that were once considered subspecies occurring on either side of the Mangoro River have since been elevated to full species status (North of the Mangoro: *P. diadema* and *E. fulvus*; South of the Mangoro: *P. edwardsi* and *E. rufifrons*: Mittermeier et al. 2010; see also Markolf and Kappeler 2013).

### Population dynamics and patterns of gene flow

Ruffed lemurs, like most mammals (Greenwood 1980), are generally assumed to exhibit female philopatry. That is, males within the species are considered the predominantly dispersing sex (Kappeler 1997; Morland 1991; but see Balko 1998; Erhart and Overdorff 2008; S. M. Holmes, S. E. Johnson, E. E. Louis, pers. obs.). By contrast, our analyses did not detect significant differences between males and females, instead indicating a lack of sex-biased dispersal in *V. variegata* (Table 4). However, simulation studies have shown that tests based on *m*AIc and *F*_ST_ can only reliably detect sex biases in dispersal when the bias is quite large and only with exhaustive sampling (Goudet et al. 2002). Although our sampling strategy was geographically extensive, sample sizes across some localities were limited.

Bottleneck analyses detected significant deviations from mutation-drift equilibrium in 50% of sites tested (5 of 10) under two (IAM, TPM) of the three models, but only two sites under the SMM. Earlier work has suggested that the TPM may be the most appropriate model for microsatellites given its intermediate status between the more conservative SMM and the rather unconstrained IAM (Di Rienzo et al. 1994; Piry et al. 1999). The general approach implemented in Bottleneck software is known to lack power simply because summary statistics do not use the genetic information very efficiently (Felsenstein 1992). Thus, if we had detected significant signals of population bottlenecks across all mutation models, this would have suggested that the signal was strong enough to be detected using a summary approach, as was found in orangutans by Goossens et al. (2006). Unfortunately, tests of excess heterozygosity have limited power with small sample sizes (Peery et al. 2012). Thus, our results should be viewed with caution, particularly among northern sampling localities. Furthermore, several studies have now shown that population structure can generate spurious bottleneck signals (e.g., Wakeley 1999; Chikhi et al. 2010). Thus, future work that seeks to identify population declines within this species will benefit from more sophisticated methods, such as those used by Olivieri et al. (2008) and Craul et al. (2009).

### Conservation applications

Results from this study have important implications for lemur conservation. Conservation genetics provides a powerful tool with which to identify important conservation priorities and also monitor the fate of populations (Schwartz et al. 2006, Frankham 2010). Effective conservation management often depends on the identification of management units (MUs), which are usually defined as demographically independent populations whose population dynamics (e.g., population growth rate) depend largely on local birth and death rates rather than on immigration. The identification of MUs is central to the short-term management and conservation of natural populations and is typically used to delineate entities for monitoring (Schwartz et al. 2006) and regulating the effects of human activity upon the abundance of populations and species. In the absence of population genetic and/or long-term demographic information, however, MUs are often identified on the basis of taxonomic (i.e., subspecies) designations to target conservation priorities and assess potential translocations and/or reintroductions (Templeton 1986; Lynch 1996).

The pattern of genetic differentiation found in this study contradicts expectations based on current taxonomy and thus calls into question the appropriateness of treating the three *Varecia* subspecies as discrete units for existing *in situ* and captive population management plans. Although they do not differ morphometrically (Baden et al. 2008), subspecies exhibit a wide variety of pelage variation (i.e., the patterning of saddles; “lightness” or “redness” of coat color) (Vasey and Tattersall 2002). There is, however, little indication that coat color pattern corresponds to either geographic location or genetic type (Wyner et al. 1999; Vasey and Tattersall 2002). Although the goal of this study was not to re-evaluate the taxonomic status of *V. variegata* subspecies, we found that the current subspecific taxonomy provides a misleading view of population differentiation (Fig. 1A and B). Both microsatellite and mtDNA sequence data grouped *V. variegata* into northern and southern genetic clusters, much like patterns identified previously by Wyner et al. (1999). We therefore propose that future conservation efforts should consider treating genetic clusters (such as those identified herein), not current subspecies, as distinct MUs, as genetic variation is arguably a more biologically accurate metric. We also hope the results presented herein will prompt a re-evaluation of the existing subspecies designations (Fig. 1).

Beyond identifying units for conservation management, this analysis has also allowed us to understand patterns of genetic diversity and thus suggest targeted conservation strategies. Until recently, northern *V. variegata* sites have likely experienced the greatest connectivity (i.e., gene flow) and genetic diversity among sampling localities; however, this is also where a majority of the illegal hunting and timber extraction has occurred due to recent political unrest (Barrett et al. 2010; Jenkins et al. 2011; Allnutt et al. 2013). *Varecia variegata* populations, among other lemur species, are currently being hunted at unsustainable levels (Golden 2009). On the other hand, animals from sampling localities within the southern cluster have significantly lower allelic, genetic, and haplotypic diversity than sites in the north. Most southern localities exhibit significant genetic differentiation (*F*_ST_), and there is some evidence that several have undergone recent population declines (Holmes et al. 2013; this study). We therefore propose that future conservation efforts should focus on maintaining genetic diversity in northern sampling localities by focusing on reducing hunting pressures and forest loss, while also increasing connectivity among southern localities to encourage gene flow among isolated populations. Ongoing efforts initiated by EEL are already underway to link fragments in Kianjavato/Vatovavy area via grassroots reforestation projects such as the Education Promoting Reforestation Project (Manjaribe et al. 2013).

Finally, our results raise the possibility of returning confiscated animals to their likely region of origin, and estimating the likely provenance of some captive animal populations. Nearly, all individuals (91%) within our study were assigned to their source localities with high statistical certainty across genetically differentiated sampling localities. Even in cases where individuals could not be assigned to their particular locality of origin, they could be successfully assigned to their appropriate genetic cluster (9%). Interestingly, Structure analyses clustered individuals from Nosy Mangabe (Nosy), an isolated *V. variegata* population originally introduced to the island in the 1930s (Petter and Peyreiras 1970), exclusively with individuals from Mananara Nord (Nara). Although, to the best of our knowledge, no known records exist regarding their true origins (I. Porton, pers. comm.), our results suggest that the founder population for the Nosy Mangabe individuals might have come from Mananara Nord, a coastal mainland site located ∼312 km south of the island. Thus, sampling animals and localities from across the full species’ range – as we have done in this study – provides not only a comprehensive picture of genetic diversity, but also useful tools for wildlife forensics.

## Data accessibility

DNA sequences have been deposited in GenBank (accessions KJ700486-KJ700626; AF495865-AF495895, AY588494, and AF493669-AF493671; see also Louis et al. 2005, 2006; Andriantompohavana et al. 2006).

## Supporting Information

**Figure S1**. Δ*K* values, a measure of the rate of change in the Structure likelihood function, as a function of K, the number of putative iterations. Results from both microsatellite (A) and mtDNA (B) analyses both indicate *K* = 2 populations.

**Figure S2**. Δ*K* values as a function of K. Results from subsequent Structure analysis within each of the *K* = 2 previously defined (A) northern and (B) southern clusters indicate further population substructure.

**Figure S3**. Results from subsequent Structure analysis within each of the *K* = 2 previously defined (A) northern and (B) southern clusters indicate further population substructure. Each animal is represented by a single horizontal bar. Locality of origin is indicated to the far left of *Q* plots. Left: Original Structure analysis (*n* = 129 adults) identifying *K* = 2 populations. Center: Subsequent Structure analysis within each of the *K* = 2 previously defined clusters (Cluster 1: North; Cluster 2: South) identifying further population substructure in both northern (*K* = 2) and southern (*K* = 2) populations. Right: Structure analysis identifying *K* = 4 clusters among the northern 8 sampling localities. The posterior probability of *K* = 4 was less than that of *K* = 2 among northern sampling localities (see Fig. S2) and was thus not selected using the Evanno et al. (2005) method.

**Table S1**. Characteristics of 10 microsatellite markers amplified in 209 *V. variegata* samples, including number of alleles per locus (*n*_*A*_), observed (*Ho*) and expected (*He*) heterozygosity, and deviations from Hardy–Weinberg Equilibrium (HWE). Significant values at *P* < 0.05 are shown in bold.

**Table S2**. Allelic diversity within each of 19 sampling localities, including mean number of alleles per locus (*MNA*), allelic richness (*AR*), observed (*Ho*) and expected (*He*) heterozygosity, inbreeding coefficient (*F*_IS_) and significant deviations from Hardy–Weinberg Equilibrium (HWE) calculated using 10,000 iterations. Significant values at *P* < 0.05 are indicated in bold. ‘-’ indicates populations with <10 samples that were not tested from deviations from HWE.

**Table S3**. Pairwise *F*_ST_ values (above diagonal) and significance values (below diagonal) among sampling localities and populations of *V. variegata*. * indicates significant values at *P* < 0.0003 after Bonferroni corrections.

**Table S4**. Measures of haplotype diversity across sampling localities, including the number of samples analyzed (*n*), the number of polymorphic sites (S), haplotype diversity (h), and nucleotide diversity (π).

**Table S5**. Pairwise Φ_PT_ values (*F*_ST_ analog; above diagonal) and significance values (below diagonal) among sampling localities and populations of *V. variegata*. * indicates significant values at *P* < 0.0003 after Bonferroni corrections.
