# Supplementary material for: Species-level view of population structure and gene flow for a critically endangered primate (Varecia variegata)
Source: Ecol Evol. 2014 Jun 6;4(13):2675–92. doi: 10.1002/ece3.1119 (PMC4113292; doi:10.1002/ece3.1119)
Supplement: Supplementary file 4 — Table S1. Characteristics of 10 microsatellite markers amplified in 209 V. variegata samples, including number of alleles per locus (nA), observed (Ho) and expected (He) heterozygosity, and deviations from Hardy–Weinberg Equilibrium (HWE). Significant values at P < 0.05 are shown in bold. [file ece30004-2675-SD4.pdf]

Table S1. Characteristics of 10 microsatellite markers amplified in 209 *V. variegata* samples, including the number of alleles per locus (n_A), observed (H_o) and expected (H_e) heterozygosity, and deviations from Hardy-Weinberg Equilibrium (HWE). Significant values at $p < 0.05$ are shown in **bold**.

Marker	Size range	Annealing temp.	n_A	H_o	H_e	HWE	GenBank	Reference
51HDZ20	221-241	50	13	0.514	0.543	0.244	AF468499	Louis et al. 2005
51HDZ25	169-175	54	5	0.45	0.498	0.083	AF468500	Louis et al. 2005
51HDZ204	129-139	60	4	0.399	0.535	**<0.001**	AF468503	Louis et al. 2005
51HDZ247	249-265	50	10	0.596	0.701	**0.001**	AF468504	Louis et al. 2005
51HDZ560	253-259	52	10	0.516	0.571	**0.021**	AF468508	Louis et al. 2005
51HDZ598	201-217	50	9	0.611	0.613	0.332	AF468509	Louis et al. 2005
51HDZ691	233-251	50	13	0.61	0.622	0.748	AF468512	Louis et al. 2005
51HDZ790	211-215	50	5	0.306	0.346	0.251	AF468513	Louis et al. 2005
51HDZ816	280-292	54	10	0.621	0.667	0.188	AF468514	Louis et al. 2005
51HDZ988	102-116	50	10	0.575	0.615	0.975	AF468519	Louis et al. 2005
