# Supplementary material for: Species-level view of population structure and gene flow for a critically endangered primate (Varecia variegata)
Source: Ecol Evol. 2014 Jun 6;4(13):2675–92. doi: 10.1002/ece3.1119 (PMC4113292; doi:10.1002/ece3.1119)
Supplement: Supplementary file 5 — Table S2. Allelic diversity within each of 19 sampling localities, including mean number of alleles per locus (MNA), allelic richness (AR), observed (Ho) and expected (He) heterozygosity, inbreeding coefficient (FIS) and significant deviations from Hardy–Weinberg Equilibrium (HWE) calculated using 10,000 iterations. Significant values at P < 0.05 are indicated in bold. ‘-’ indicates populations with <10 samples that were not tested from deviations from HWE. [file ece30004-2675-SD5.pdf]

Table S2. Allelic diversity within each of 19 sampling localities, including mean number of alleles per locus (MNA), allelic richness (AR), observed (H_O) and expected (H_S) heterozygosity, inbreeding coefficient (F_{IS}), and significant deviations from Hardy-Weinberg Equilibrium (HWE) calculated using 10 000 iterations

Site	Geographic region	N	MNA	AR (SE)	H_O	H_S	F_{IS}	HWE
Nosy Mangabe S.R.†	North	9	3.3	1.99 (0.165)	0.367	0.478	0.233	-
Marotandrano S.R.	North	9	4.2	2.50 (0.178)	0.556	0.672	0.173	-
Mananara Nord N.P.	North	8	4.0	2.40 (0.128)	0.575	0.634	0.093	-
Ambatovaky S.R.	North	5	3.4	2.39 (0.192)	0.400	0.640	0.375	-
Zahamena N.P., S.N.R.	North	10	4.7	2.52 (0.182)	0.620	0.658	0.058	0.167
Betampona S.N.R.	North	9	3.6	2.26 (0.140)	0.544	0.594	0.083	-
Mangerivola S.R.	North	3	3.3	2.61 (0.275)	0.633	0.667	0.050	-
Mantadia Andasibe N.P.	North	14	3.7	2.24 (0.194)	0.514	0.573	0.103	0.061
Torotorofotsy	North	3	2.5	2.10 (0.245)	0.533	0.483	-0.103	-
Maromizaha U.F.	North	2	2.2	2.20 (0.291)	0.700	0.500	-0.400	-
Anosibe an'ala C.F.	North	8	3.5	2.31 (0.183)	0.493	0.602	0.181	-
Fandriana U.F.	South	11	4.5	2.33 (0.161)	0.536	0.609	0.119	**0.030**
Vatoharanana (Ranomafana N.P.)	South	10	3.6	2.20 (0.149)	0.490	0.566	0.134	**0.035**
Mangevo (Ranomafana N.P.)	South	30	3.9	2.41 (0.110)	0.627	0.648	0.032	0.215
Kianjavato U.F.	South	32	3.6	1.95 (0.161)	0.459	0.460	0.001	0.500
Vatovavy U.F.	South	21	2.9	1.97 (0.132)	0.468	0.487	0.038	0.293
Lakia	South	10	2.6	2.04 (0.110)	0.460	0.527	0.128	0.062
Tolongoina U.F.	South	4	2.8	2.12 (0.268)	0.400	0.504	0.207	-
Manombo S.R.	South	11	2.7	2.00 (0.149)	0.500	0.488	-0.025	0.388
Overall		209	10.2	2.25 (0.182)	0.519	0.573	0.094	

N = no. samples successfully analyzed; MNA = mean number of alleles per locus; AR = Allelic diversity after correcting for sample size with rarefaction (standard error); H_O = observed heterozygosity; H_S = expected heterozygosity assuming HWE corrected for sampling bias (Simpson's Diversity Index); F_{IS} = inbreeding coefficient; HWE = one-sided *p* calculated using 10 000 permutations. Significant values indicated in **bold**. '-' indicates populations with < 10 samples that were not tested for Hardy-Weinberg Equilibrium.
