# Supplementary material for: Species-level view of population structure and gene flow for a critically endangered primate (Varecia variegata)
Source: Ecol Evol. 2014 Jun 6;4(13):2675–92. doi: 10.1002/ece3.1119 (PMC4113292; doi:10.1002/ece3.1119)
Supplement: Supplementary file 6 — Table S3. Pairwise FST values (above diagonal) and significance values (below diagonal) among sampling localities and populations of V. variegata. * indicates significant values at P < 0.0003 after Bonferroni corrections. [file ece30004-2675-SD6.pdf]

Table S3. Pairwise F_{ST} values (above diagonal) and significance values (below diagonal) among sampling localities and populations of *V. variegata*. Significant values indicated with * (P < 0.0003 after Bonferroni corrections)

		Northern sites										
		**NosyMangabe**	**Marontandrano**	**Mananara Nord**	**Ambatovaky**	**Zahamena**	**Betampona**	**Mangerivola**	**Mantadia**	**Torotorofotsy**	**Maromizaha**	**Anosibe**
**Northern sites**	**NosyMangabe**	--	0.203	0.127	0.161	0.194	0.225	0.179	0.291	0.361	0.383	0.307
	**Marontandrano**	*	--	0.092	0.080	0.038	0.091	0.065	0.174	0.221	0.205	0.189
	**MananaraNord**	NS	NS	--	0.094	0.111	0.130	0.129	0.209	0.270	0.257	0.243
	**Ambatovaky**	NS	NS	NS	--	0.084	0.091	0.002	0.114	0.146	0.132	0.168
	**Zahamena**	*	NS	*	NS	--	0.137	0.090	0.138	0.176	0.156	0.148
	**Betampona**	*	NS	*	NS	*	--	0.035	0.119	0.233	0.201	0.167
	**Mangerivola**	NS	NS	NS	NS	NS	NS	--	0.071	0.162	0.139	0.090
	**Mantadia**	*	*	*	NS	*	*	NS	--	0.051	0.055	0.068
	**Torotorofotsy**	NS	NS	NS	NS	NS	NS	NS	NS	--	0.120	0.133
	**Maromizaha**	NS	NS	NS	NS	NS	NS	NS	NS	NS	--	0.067
	**Anosibe**	*	*	*	*	*	*	NS	NS	NS	NS	--
	**N**	9	9	8	5	10	9	3	14	3	2	8

		Southern sites							
		**Fandriana**	**Vato**	**Mangevo**	**Kianjavato**	**Vatovavy**	**Lakia**	**Tolongoina**	**Manombo**
**Southern sites**	**Fandriana**	--	0.145	0.104	0.203	0.155	0.190	0.189	0.213
	**Vato**	*	--	0.089	0.284	0.241	0.282	0.235	0.278
	**Mangevo**	*	*	--	0.241	0.179	0.193	0.227	0.206
	**Kianjavato**	*	*	*	--	0.176	0.123	0.339	0.287
	**Vatovavy**	*	*	*	*	--	0.160	0.251	0.269
	**Lakia**	NS	NS	*	*	*	--	0.292	0.222
	**Tolongoina**	*	*	*	*	*	NS	--	0.297
	**Manombo**	*	*	*	*	*	NS	*	--
	**N**	11	10	30	32	21	10	4	11
