# Supplementary material for: Species-level view of population structure and gene flow for a critically endangered primate (Varecia variegata)
Source: Ecol Evol. 2014 Jun 6;4(13):2675–92. doi: 10.1002/ece3.1119 (PMC4113292; doi:10.1002/ece3.1119)
Supplement: Supplementary file 7 — Table S4. Measures of haplotype diversity across sampling localities, including the number of samples analyzed (n), the number of polymorphic sites (S), haplotype diversity (h), and nucleotide diversity (π). [file ece30004-2675-SD7.pdf]

Table S4. Measures of haplotype diversity across sampling localities, including the number of samples analyzed (n), the number of polymorphic sites (S), haplotype diversity (h), and nucleotide diversity (*π*)

Site	Geographic region	n	S	No. haplotypes	h	*π*
Nosy Mangabe S.R.†	North	9	1	2	0.2222	0.0004
Marotandrano S.R.	North	9	5	2	0.3889	0.0035
Mananara Nord N.P.	North	8	3	2	0.2500	0.0013
Ambatovaky S.R.	North	5	3	2	0.4000	0.0021
Zahamena N.P., S.N.R.	North	11	19	3	0.6364	0.0172
Betampona S.N.R.	North	9	20	2	0.3889	0.0139
Mangerivola S.R.	North	3	0	1	0.0000	0.0000
Mantadia Andasibe N.P.	North	14	9	2	0.4945	0.0080
Torotorofotsy	North	3	9	2	0.6667	0.0107
Maromizaha U.F.	North	4	0	1	0.0000	0.0000
Anosibe an'ala C.F.	North	8	0	1	0.0000	0.0000
Fandriana U.F.	South	11	0	1	0.0000	0.0000
Vatoharanana (Ranomafana N.P.)	South	10	0	1	0.0000	0.0000
Mangevo (Ranomafana N.P.)	South	8	0	1	0.0000	0.0000
Kianjavato U.F.	South	12	0	1	0.0000	0.0000
Vatovavy U.F.	South	10	0	1	0.0000	0.0000
Lakia	South	10	0	1	0.0000	0.0000
Tolongoina U.F.	South	4	0	1	0.0000	0.0000
Manombo S.R.	South	11	0	1	0.0000	0.0000
Overall		159	44	19	0.8557	0.0190

n = no. samples analyzed; S = no. polymorphic sites; h = haplotype diversity; π = nucleotide diversity
