# Supplementary material for: Species-level view of population structure and gene flow for a critically endangered primate (Varecia variegata)
Source: Ecol Evol. 2014 Jun 6;4(13):2675–92. doi: 10.1002/ece3.1119 (PMC4113292; doi:10.1002/ece3.1119)
Supplement: Supplementary file 8 — Table S5. Pairwise ΦPT values (FST analog; above diagonal) and significance values (below diagonal) among sampling localities and populations of V. variegata. * indicates significant values at P < 0.0003 after Bonferroni corrections. [file ece30004-2675-SD8.pdf]

Table S5. Pairwise PhiPT values (FST analog; above diagonal) and significance values (below diagonal) among sampling localities and populations of V. variegata. Significant values indicated with * (P < 0.003 after Bonferroni corrections)

		Northern sites										
		NosyMangabe	Marontandrano	MananaraNord	Ambatovaky	Zahamena	Betampona	Mangerivola	Mantadia	Torotorofotsy	Maromizaha	Anosibe
Northern sites	Nosy Mangabe	--	0.8425	0.9203	0.9816	0.7594	0.8387	0.9926	0.8696	0.9270	0.9933	0.6834
	Marontandrano	*	--	0.7190	0.9245	0.6774	0.7779	0.9124	0.7834	0.8022	0.9016	0.6216
	MananaraNord	*	NS	--	0.9570	0.7130	0.7988	0.9664	0.8352	0.8810	0.9669	0.6451
	Ambatovaky	*	NS	NS	--	0.5191	0.5280	0.9635	0.7448	0.7919	0.9567	0.5456
	Zahamena	*	*	*	NS	--	0.1117	0.5461	0.4925	0.3340	0.4601	0.4310
	Betampona	*	*	*	NS	NS	--	0.7119	0.6577	0.5574	0.6662	0.5005
	Mangerivola	NS	NS	NS	NS	NS	NS	--	0.6393	0.6857	0.0000	0.4003
	Mantadia	*	*	*	*	*	*	NS	--	0.0000	0.2628	0.3985
	Torotorofotsy	NS	NS	NS	NS	NS	NS	NS	NS	--	0.1111	0.1930
	Maromizaha	NS	NS	NS	NS	NS	NS	NS	NS	NS	--	0.4049
	Anosibe	*	*	NS	*	*	*	NS	*	NS	NS	--
	N	9	9	8	5	11	9	3	14	3	4	8

		Southern sites							
		Fandriana	Vato	Mangevo	Kianjavato	Vatovavy	Lakia	Tolongoina	Manombo
Southern sites	Fandriana	--	0.0000	0.0000	0.0000	0.0000	0.0000	0.0000	0.0000
	Vato	NS	--	0.0000	0.0000	0.0000	0.0000	0.0000	0.0000
	Mangevo	NS	NS	--	0.0000	0.0000	0.0000	0.0000	0.0000
	Kianjavato	NS	NS	NS	--	0.0000	0.0000	0.0000	0.0000
	Vatovavy	NS	NS	NS	NS	--	0.0000	0.0000	0.0000
	Lakia	NS	NS	NS	NS	NS	--	0.0000	0.0000
	Tolongoina	NS	NS	NS	NS	NS	NS	--	0.0000
	Manombo	NS	NS	NS	NS	NS	NS	NS	--
	N	11	10	8	12	10	10	4	11
